# Hexagonal Boron Nitride as Filler for Silica-Based Elastomer Nanocomposites

**DOI:** 10.3390/nano14010030

**Published:** 2023-12-21

**Authors:** Federica Magaletti, Gea Prioglio, Ulrich Giese, Vincenzina Barbera, Maurizio Galimberti

**Affiliations:** 1 Department of Chemistry, Materials and Chemical Engineering “G. Natta”, Politecnico di Milano, Via Mancinelli 7, 20131 Milano, Italy; federica.magaletti@polimi.it (F.M.); gea.prioglio@polimi.it (G.P.); 2 Deutsches Institut für Kautschuktechnologie e. V., Eupener Straße 33, 30519 Hannover, Germany

**Keywords:** rubber compounds, 2D nanomaterials, h-BN functionalization, lower Payne effect

## Abstract

Two-dimensional hexagonal boron nitride (hBN) has attracted tremendous attention over the last few years, thanks to its stable structure and its outstanding properties, such as mechanical strength, thermal conductivity, electrical insulation, and lubricant behavior. This work demonstrates that hBN can also improve the rheological and mechanical properties of elastomer composites when used to partially replace silica. In this work, commercially available pristine hBN (hBN-p) was exfoliated and ball-mill treated in air for different durations (2.5, 5, and 10 h milling). Functionalization occurred with the -NH and -OH groups (hBN-OH). The functional groups were detected using Fourier-Transform Infrared pectroscopy (FT-IR) and were estimated to be up to about 7% through thermogravimetric analysis. The presence of an increased amount of oxygen in hBN-OH was confirmed using Scanning Electron Microscopy coupled with Energy-Dispersive X-ray Spectroscopy. (SEM-EDS). The number of stacked layers, estimated using WAXD analysis, decreased to 8–9 in hBN-OH (10 h milling) from about 130 in hBN-p. High-resolution transmission electron microscopy (HR-TEM) and SEM-EDS revealed the increase in disorder in hBN-OH. hBN-p and hBN-OH were used to partially replace silica by 15% and 30%, respectively, by volume, in elastomer composites based on poly(styrene-co-butadiene) from solution anionic polymerization (S-SBR) and poly(1,4-cis-isoprene) from *Hevea Brasiliensis* (natural rubber, NR) as the elastomers (volume (mm^3^) of composites released by the instrument). The use of both hBNs in substitution of 30% of silica led to a lower Payne effect, a higher dynamic rigidity, and an increase in E′ of up to about 15% at 70 °C, with similar/lower hysteresis. Indeed, the composites with hBN-OH revealed a better balance of tan delta (higher at low temperatures and lower at high temperatures) and better ultimate properties. The functional groups reasonably promote the interaction of hBN with silica and with the silica’s coupling agent, sulfur-based silane, and thus promoted the interaction with the elastomer chains. The volume of the composite, measured using a high-pressure capillary viscometer, increased by about 500% and 400% after one week of storage in the presence of hBN-p and hBN-OH. Hence, both hBNs improved the processability and the shelf life of the composites. Composites obtained using hBN-OH had even filler dispersion without the detachments of the filler from the elastomer matrix, as shown through TEM micrographs. These results pave the way for substantial improvements in the important properties of silica-based composites for tire compounds, used to reduce rolling resistance and thus the improve environmental impacts.

## 1. Introduction

Since the discovery of graphene [1] and the micromechanical isolation of single-layered graphene (the scotch tape experiment) [2], research in the field of two-dimensional (2D) nanomaterials has exponentially grown [3,4,5,6,7,8]. Their atomic thickness, their very high area/volume ratio, their significant potential for applications in performing functionalization reactions, and their remarkable physical, electrical, and optical properties have captivated researchers from diverse domains; such domains include condensed matter physics, materials science, chemistry, and nanotechnology. Research in these fields has paved the way for a variety of applications, from the biomedical field [9] to gas sensing [10] to the tailoring of friction and wear in machine elements [11,12,13]. 

Among all the studied 2D materials, boron nitride (BN) is an extremely versatile material. BN is a ceramic material with an equal number of boron (B) and nitrogen (N) atoms. Different allotropic forms can be described: cubic BN (cBN), wurstzite BN (wBN), hexagonal boron nitride (h-BN), and turbostratic BN [14].

h-BN is the most stable allotropic form of BN and is a synthetic material. It is also called graphitic boron nitride and white graphene [15], due to its similarity to the sp^2^ carbon allotrope homologue. h-BN is a layered material and is characterized by high hardness, high mechanical strength, low roughness, good thermal stability, good thermal conductivity, electrical insulation, lubricant properties, and good biocompatibility [16,17,18,19,20,21,22,23]. As a consequence, h-BN is the most widely used BN allotrope, and significant interest in this material has been developing over recent years; there are many, varied potential applications for it [24,25,26,27,28,29,30,31,32,33,34,35].

The 2030 Agenda for Sustainable Development incorporates sustainable transportation into multiple Sustainable Development Goals [36]. The energy sector is the most significant contributor to global greenhouse gas emissions (more than 70%); road transport is the most impactful sector in this contribution, accounting for about 12%. Tires play a key role in sustainable development. The worldwide tire market is expected produce 2.7 billion units in 2025 [37]. The rolling resistance (RR), defined as “the energy consumed per unit distance of travel as a tire rolls under load” [38], holds the primary responsibility for a tire’s environmental impact during its use. Tire compounds must be designed in order to reduce their hysteresis and thus the rolling resistance. Nowadays, in tire compounds, precipitated silica [39,40] is the preferred filler for preparing materials with low hysteresis [39,40,41]. The silanol groups on its surface allow reactions to occur with sulfur-containing silanes, which act as coupling agents with the elastomer matrix through crosslinking reactions [42]. The chemical bond between silica and the polymer chains plays a key role in reducing the composite’s hysteresis. However, silica has many technical drawbacks. Some of them arise from the high surface activity of silica, which promotes extensive supramolecular interactions. Silica causes increases in the viscosity of the elastomer composite, and leads to decreased processability and reduced storage time. The short storage time has a clear impact on planning for the production and transportation of the composites; in turn, there are consequences for the logistics. Ad hoc mixing equipment that is more expensive than regular equipment must be used to ensure the efficient processing of silica-based composites. The silane increases the adherence of the material to the metal parts of the mixing machines. Moreover, silica is corrosive and abrasive. Hence, special treatments of the metal surfaces and particular revision procedures must be performed. All these technical problems are remarkable at the industrial scale, in the light of the great number of tires on the market. Thus, it would be highly desirable to achieve improved (or at least the same) dynamic mechanical properties for silica-based composites; this might be possible through using a lower amount of silica; this would reduce—at least to some extent—the mentioned technical drawbacks. 

To this aim, an interesting potential approach is the partial replacement of silica with a filler that is suitable for interrupting the 3D network and weakening the supramolecular interaction. From this perspective, 2D nanomaterials are of great interest. Researchers have recently reported on the use of 2D graphene nanoplatelets (GnP) in partial replacement of silica, obtaining similar dynamic mechanical characteristics and better rheological properties in comparison with the original material [43]. Due to the poor compatibility of GnP with silica, flaws were found in the elastomer composite and only the introduction of polar groups on the GnP edges, through the functionalization with a pyrrole compound such as 2-(2,5-dimethyl-1*H*-pyrrol-1-yl)propane-1,3-diol (serinol pyrrole), allowed the researchers to obtain a material with a homogenous, continuous structure. However, the functionalization method required polluting steps, with the use of an organic solvent such as acetone and of high temperature (180 °C). hBN, as a 2D material, presents advantages with respect to GnP: lower numbers of stacked layers could promote higher efficiency—the composite ingredients and the chemical nature of hBN could favor its compatibility with silica and/or its chemical modification. 

In this work, elastomer composites based on poly(styrene-co-butadiene) from anionic solution polymerization (solution styrene butadiene rubber, S-SBR) and poly(1,4-*cis*-isoprene) from *Hevea Brasiliensis* (natural rubber, NR) were prepared, with precipitated silica as the reinforcing filler. Bis(triethoxysilylpropyl)tetrasulfide (TESPT) was the organosulfur compound that was used as the coupling agent for silica with elastomer chains. Pristine hexagonal boron nitride (hBN-p) and the functionalized derivative were used in partial replacement of silica at two different levels, 15% vol and 30% vol, while maintaining the same total volume fraction of the filler. The functionalization of h-BN was performed through a simple and environmentally friendly mechanical approach: ball milling [44]. In previous studies, the functionalization of hBN has been performed with various methods [45,46,47,48,49,50,51,52,53,54,55]. Mechanochemistry was applied using sonication [47,48] and ball milling [49,50,51]. For the introduction of hydroxyl groups, hBN was treated with air plasma [52], steam (Ar flow) at 850 °C and 1000 °C [53], and hot aqueous solution of H_2_SO_4_/KMnO_4_ [54]. Ball milling was performed in the presence of water solutions of either NaOH [49] or NaOH/KOH [50]. Sonication was carried out at room temperature in the presence of H_2_O [47] or N-methylpyrroliperformed [48].

In this work, functionalization was used to enable the introduction of OH groups, relying on the Lewis’ base character of hBN; hence, milder and scalable experimental conditions were used, without the need for any chemical substance. Mechanical treatment of hBN was performed through ball milling at a nominal room temperature in air; the samples taken from the jar were washed with water. The functionalized hBN is referred to in this paper as hBN-OH. hBN-p and hBN-OH, as taken from the milling jar and after washing with water, were characterized through thermogravimetric analysis (TGA), Fourier-transform infrared spectroscopy (FT-IR), wide-angle X-ray diffraction (WAXD), and high-resolution transmission electron microscopy (HR-TEM). The elastomer composites were prepared via melt blending in an internal mixer; they were additionally crosslinked using a system based on sulfur and a sulphenamide, such as *N*-tert-butyl-2-benzothiazyl sulfenamide (TBBS). They were characterized through studying the crosslinking reaction and determining the dynamic mechanical properties in both the shear and the axial modes; the tensile properties were also studied. Their structure was analyzed through TEM. The composites were extruded from a high-pressure capillary viscometer (HKV) to investigate their processability.

## 2. Materials and Methods

### 2.1. Materials

#### 2.1.1. Chemicals

All reagents and solvents were purchased and used without further purification: acetone was obtained from Sigma-Aldrich. 

The following chemicals were used in the preparation of the elastomeric composites: Bis(triethoxysilylpropyl)tetrasulfide (TESPT; Evonik Industries, Essen, Germany), ZnO (Zincol Ossidi, Bellusco, MB, Italy), stearic acid (Sogis, Milan, Italy), 1,3-dimethyl butyl)-*N*′-Phenyl-p-phenylenediamine (6PPD from Eastman Kingsport, TN, USA), sulfur (S from Solfotecnica, Cotignola, Italy), and *N*-tert-butyl-2-benzothiazyl sulfenamide (TBBS from Lanxess Chemical, Shangai, China).

#### 2.1.2. Elastomeric Materials 

Natural rubber (poly(1,4-*cis*-isoprene) from Hevea brasiliensis) was purchased from EQR-E.Q. Rubber, BR-THAI, Eastern GR. Thailand—Chonburi, with the trade name SIR20. The rubber has a Mooney viscosity (ML (1 + 4) 100 °C) of 73 MU. Solution styrene butadiene, not functionalized (SPRINTAN™ SLR 4630 from Trinseo, Milano, Italy), was employed. The composition of the S-SBR was as follows: Stirene 25%, Butadiene 75% and 37.5 phr of TDAE. The Butadiene fraction has a vinyl content of 63%. Other properties of S-SBR are Tg-29C, not functionalized. Mooney viscosity (ML (1 + 4) 100 °C) of 55 MU.

#### 2.1.3. Fillers

Hexagonal boron nitride industrial-grade A01 was kindly provided by Hoganas (Hoganas, Sweden). Data from the technical data sheet are as follows: Boron (42.5–43.5%); Oxigen 1.2% B_2_O_2_ (water soluble), H_2_O, and C less than 0.15% each, Cristallographic Phases Hexagonal, high degree of crystallization. Specific surface area (NSA): 3.5–7.0 m^2^/g. Tap density: 0.2–0.5 g/cm^3^.

Silica ZEOSIL 1165MPmicropearl silica was purchased from Solvay (Brussels, Belgium). The main properties of the material, reported in the technical data sheet, were as follows: loss on drying (2 h @ 105 °C) ≤ 8.0%; soluble salts (as Na_2_SO_4_) ≤ 2.0%; specific surface area—140–180 m^2^/g.

The BET method was used to determine the surface area through the use of a MICROACTIVE TRISTAR^®^ II PLUS apparatus. Samples were evacuated at 200 °C for 2 h and N_2_ adsorption isotherms were recorded at 77 K in a liquid nitrogen bath. The specific surface area (SSA) was found to be 160 m^2^/g.

### 2.2. Preparation of Functionalized Boron Nitride (hBN-OH)

The white powder of hBN-p was treated using a planetary ball mill S100 from Retsch (Haan, Germany); the zirconia grinding jar moved in a horizontal plane with a volume of 0.3 L. The powder was previously kept closed in a PET jar in a room at 19 °C and a relative humidity of 47%. The grinding jar was loaded with 7 ceramic balls with a diameter of 15 mm. A measurement of 5 g of hBN-p was put into the jar at 19 °C and a relative humidity of 47%, without any liquids. Once the jar was closed, it was made to rotate at 300 rpm at nominal room temperature in air. Milling was carried out for 2.5, 5, and 10 h. When the jar was opened, the odor of ammonia was present; this was faint following 2.5 h milling and strong following 10 h milling. Since the powder was grinded without liquids, the process can be seen as a dry grinding process.

After milling, the powder was placed in a becher and washed with 300 mL of deionized water and stirred for 4 h at 300 rpm. The mixture was then filtered on a Büchner funnel with a sintered glass disc. The wet powder was finally dried in the air. A measure of 5 g of the dark-grey powder was collected.

### 2.3. Preparation of Elastomeric Composites 

Elastomeric nanocomposites were prepared, with S-SBR 4630 as the main rubber (70 phr), and with NR (30 phr). Precipitated silica was the filler (50 phr). Partial replacement of silica at 15% vol and 30% vol was performed with either pristine hBN or with hBN-OH obtained through a 5 h ball-milling process followed by washing with water. Sulfur-based crosslinking was performed using a typical recipe that was suitable for the crosslinking of S-SBR. In particular, a relatively low amount of ZnO was used. The recipes of the composites can be found in Table 1. 

**Figure 1:**
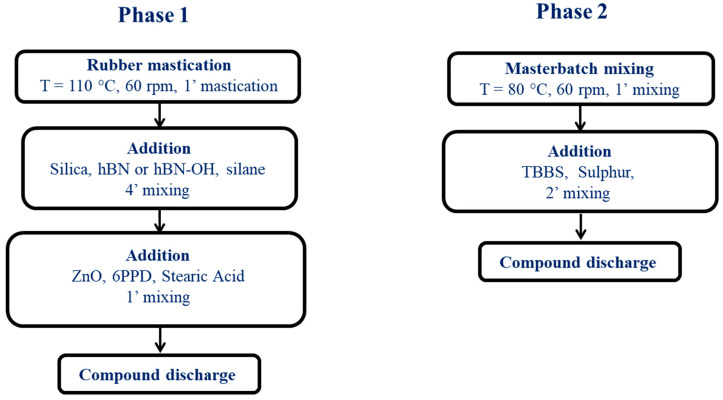
Block scheme of the mixing procedure.

Mixing was performed as summarized in Figure 1. 

The composites were prepared using a Brabender^®^ internal mixer with a 55 cc chamber (Torque Rheometer Brabender^®^ PL-2000 Plasti-Corder, from Brabender GmbH & Co. KG, Duisburg, Germany). NR and S-SBR were fed into the mixer and masticated at 110 °C and 60 rpm for 60 s. Then, silica, either hBN-p or hBN-OH, and the silane TESPT were added and mixing was performed for 4 min. ZnO, 6PPD, and stearic acid were added, and mixing was performed for 1 min. The composite was then discharged and was then fed again to the mixer at 80 °C, performing mixing for 1 min, followed by the addition of the vulcanizers. The compound was discharged after 2 min of mixing. The composites were lastly homogenized, employing a two-roll mill at 50 °C. Each compound was passed through the mill 5 times; the nip between the rolls was set to 1 cm, the rotation of the front roll was set to 30 rpm, and the rotation of the back roll was set to 38 rpm.

### 2.4. Characterization Techniques

#### 2.4.1. Thermogravimetric Analysis

TGA tests were performed through heating samples (10 mg) from 30 to 900 °C with a heating rate of 10 °C/min in air (60 mL/min); these were carried out using a Mettler TGA SDTA/851 instrument following the ISO9924-1 standard [56]. 

TGA was used to estimate the extent of functionalization, which was considered to be the mass loss% in the T range from 150 °C to 900 °C. 

#### 2.4.2. Fourier-Transform Infrared (FT-IR)

FT-IR absorption spectra were recorded in transmission mode using a FT-IR Nicolet Nexus spectrometer coupled with a ThermoElectron FT-IR Continuµm IR microscope (Thermo Fisher Scientific, Waltham, MA, USA) (resolution: 4 cm^−1^; scans: 30). Attenuated total reflectance ATR was carried out with a Si tip single bounce slide on an ATR accessory in a spectral range between 4000 cm^−1^ and 700 cm^−1^. Analysis of FTIR spectra was conducted using Origin Pro 2018

#### 2.4.3. Wide-Angle X-ray Diffraction (WAXD)

WAXD was performed with a Bruker D8 Advance automatic diffractometer (Bruker, Billerica, MA, USA)and nickel-filtered Cu-Kα radiation; wide-angle X-ray diffraction patterns were carried out in reflection. A range of 4.7° to 90° was used to record the patterns because these angles were 2θ at the peak diffraction angles.

Spectra were developed using Origin Pro 2018.

Through the Bragg law (Equation (1)), the distance between crystallographic planes was estimated.

(1) $$d_{hkl}=\frac{n\cdot \lambda }{2\cdot \sin\theta_{hkl}}$$

where n is an integer number, λ is the wavelength of the irradiating beam, and θ_hkℓ_ is the diffraction angle.

The Scherrer equation, Equation (2), was used to calculate the D_hkl_ crystallite dimensions.

(2) $$D_{hkl}=\frac{k\cdot ʎ}{\beta_{hkl}\cdot \cos\theta_{hkl}}$$

where β_hkl_ is the width at half height, θ_hkl_ is the diffraction angle, K is the Scherrer constant, and λ is the wavelength of the irradiating beam.

The number of layers in a crystallite was calculated with Equation (3) by dividing the crystallite size by the interlayer distance. In this manuscript, Equation (2) was applied to the (002) reflection.

(3) $$number\;of\;layers=\frac{D_{hkl}}{d_{hkl}}$$

#### 2.4.4. Crosslinking

Crosslinking was performed in a rubber process analyzer (Monsanto R.P.A. 2000, Alpha Thechnologies, Milano, Italy). A measure of 5 g of rubber composite was weighed and put in the rheometer. Measurements were carried out at a frequency of 1.7 Hz and an oscillation angle of 6.98%. The sample, loaded at 50 °C, underwent a first strain sweep (0.2–25% strain) to cancel the thermo-mechanical history of the rubber composite; it was then maintained at 50 °C for 10 min and then underwent another strain sweep at 50 °C to measure the dynamic mechanical properties at low deformations of the uncured sample. The crosslinking reaction was then carried out at 170 °C for 20 min. A torque–time curve was obtained. The minimum achievable torque (M_L_), the maximum achievable torque (M_H_), the time required to have a torque equal to M_L_ + 1 (t_S1_), and the time required to reach 90% of the maximum torque (t_90_) were measured. 

#### 2.4.5. Dynamic Mechanical Analysis in the Shear Mode—Strain Sweep Test

Samples for the strain sweep test were prepared as shown in the infographic in Figure S1 in the Supplementary Materials. The uncured composites were taken from the internal mixer and were processed on a two-roll mill as described in Section 2.3 and were then placed in the rheometer.

The shear dynamic mechanical characteristics of the rubber compounds were evaluated by performing strain sweep tests in a rubber process analyzer (Monsanto R.P.A. 2000 Alpha Thechnologies, Milano, Italy). As reported in Section 2.4.3, the crude sample was subjected to a first strain sweep and held at 50 °C for ten minutes, followed by another strain sweep at 50 °C. Data from the second strain sweep were collected and are reported in the text below to discuss the behavior of the uncured samples. The crosslinking was then carried out as reported in Section 2.4.4; the shear dynamic mechanical properties of the cured samples were then assessed after 20 min at 50 °C using a 0.2–25% strain sweep at a frequency of 1 Hz. The minimum strain was 0.2%, as it was the minimum required strain value to obtain reliable data from the RPA instrument. Shear storage and loss moduli (G′, G″), and subsequently Tan δ, were the measured characteristics.

#### 2.4.6. Dynamic Mechanical Analysis in the Axial Mode

Samples were prepared as shown in the infographic in Figure S1. After the processing on the two-roll mill, described in Section 2.3, the elastomeric compound was rolled up to obtain a long cylinder. This cylinder was then cut into smaller cylinders and vulcanized (at 170 °C for 20 min) to produce cylindrical test pieces with dimensions of 25 mm in length and 12 mm in diameter. An Instron dynamic device, set to the traction–compression mode, was employed to perform the dynamic mechanical measurements; this was maintained at the predetermined temperatures (10, 23, and 70 °C) throughout the entire experiment. The cylinder was preloaded to a 25% longitudinal deformation with respect to the original length. The compression was subjected to a dynamic sinusoidal strain in compression with an amplitude of around 3.5% regarding the length under pre-load, at a frequency of 100 Hz. The values of dynamic storage modulus (E′), loss modulus (E″), and loss factor (tan δ) were calculated as the ratio between E″ and E′.

#### 2.4.7. Tensile Test

Standard dumbbells made from vulcanized compound plates measuring 10 cm by 10 cm by 1 mm were used to perform the tensile tests at room temperature with a Zwick Roell Z010 (Genova, Italy) and an optical extensometer. Measurements were performed at 1 mm/min. Stresses at different elongations (σ_50_, σ_100_, and σ_300_), stress at break (σ_B_), elongation at break (ε_B_), and the energy required to break were measured according to Standard ISO 37/UNI 6065 [57].

#### 2.4.8. High-Pressure Capillary Viscosimeter (HKV)

HKV measurements were performed through a high-pressure capillary viscosimeter, type “Rheo-Vulkameter 78.90” (Göttfert Werkstoff-Prüfmaschinen GmbH, Buchen, Germany). The crude compound material was extruded using a 20 mm capillary with a 2 mm diameter at a pressure of 130 bar. The mold temperature was 100 °C. To obtain the correct experimental conditions, a preheating step of 180 s and an injection step of 60 s were carried out.

#### 2.4.9. High-Resolution Transmission Electron Microscopy

*hBN samples:* TEM and HRTEM analyses were performed with a Philips CM 200 (Eindhoven, the Netherlands) field emission gun microscope operating at an accelerating voltage of 200 kV. A few drops of a 1 mg/mL ethyl acetate dispersion were deposited on a 200-mesh lacey carbon-coated copper grid and air-dried for several hours before analysis. 

The samples were prepared with a dispersion of 1 mg/mL in ethyl acetate then sonicated to homogenize the dispersion.

*Rubber nanocomposites:* TEM characterizations were performed with a TEM, LIBRA^®^ 120, Zeiss, Oberkochen (Baden-Württemberg, Germany), with an acceleration voltage of 120 kV. Layers of vulcanized rubber were cut of a thickness of approximately 100 nm under liquid nitrogen, using a diamond cutter mounted on a Leica Ultramicrotome UC6, (Leica Microsystem, Wetzlar, Germany) equipped with a stereo microscope MZ6 and a FC6 cutting system. 

#### 2.4.10. Scanning Electron Microscope Coupled with Energy-Dispersive X-ray Spectroscopy (SEM-EDS)

The SEM-EDS investigation was carried out using a Zeiss EVO 50 EP SEM (Zeis Vision Care, Castiglione Olana, Italy) coupled with an EDS spectrometer (Bruker Quantax 200 6/30, Bruker, Billerica, MA, USA). hBN powders were applied on aluminum stubs with the aid of a conductive carbon bioadhesive. The samples were subsequently coated with gold using an Ewards S150B sputter coater (Perkin Elmer, Milan, Italy) and evaluated.

## 3. Results and Discussion

### 3.1. Preparation of hBN Samples

Pristine hBN was commercially available. The functionalization of hBN-p was attempted through ball-milling the hBN-p powder, as described in the experimental section. In brief, the powder was treated in air in a planetary ball mill at nominal room temperature and for different durations: 2.5, 5, and 10 h. All the collected powders were washed with water and filtered. The wet powder was dried for 48 h in air and then for 8 h in oven at 90 °C, under atmospheric pressure. Samples taken from the ball-milling jar—these are named, for the sake of simplicity, as hBN-OH in the paper—were characterized as such (hBN-OH_as_); then, they were washed with water (hBN-OH_w_). Characterization was performed through means of TGA, FT-IR, WAXD, and HR-TEM. 

**Figure 2:**
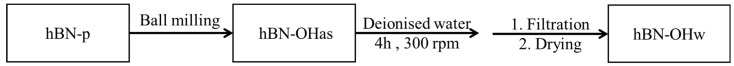
Block diagram describing the preparation of the hBN-OH samples as taken from the ball-milling jar (hBN-OH_as_) and after washing with water (hBN-OH_w_).

In Figure 2, a schematic representation of the procedure for the preparation of the hBN-OH samples is shown.

### 3.2. Characterization of hBN Samples

**Figure 3:**
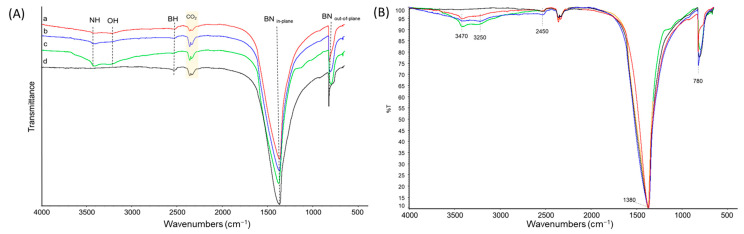
FT-IR spectra of hBN-OH_as_, milled for 2.5 (a), 5 (b), and 10 (c) h; hBN-p (d). (**A**) Spectra are displayed with normalized intensity. CO_2_ absorption bands are labeled. (**B**) Spectra are displayed after baseline correction and after normalization of the scale.

**Figure 4:**
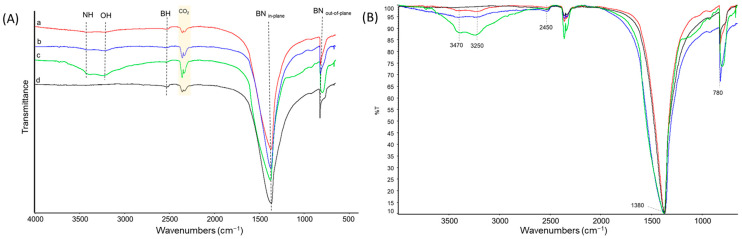
FT-IR spectra of hBN-OH_w_, milled for 2.5 (a), 5 (b), and 10 (c) h; hBN-p (d). (**A**) Spectra are displayed with normalized intensity. CO_2_ absorption bands are labeled. (**B**) Spectra are displayed after baseline correction and after normalization of the scale.

*FT-IR analysis:* The presence of functional groups on hBN samples, after ball milling and then after washing with water, was investigated through FT-IR vibrational spectroscopy. For the sake of clarity, the spectra of hBN-OH_as_ and hBN-OH_w_, milled for 2.5, 5, and 10 h, were organized in two different figures: Figure 3 and Figure 4, respectively. The proposed assignment of the main peaks of spectra of Figure 3 and Figure 4 is reported in Table 2 and Table 3, which show the relative abundances (%) of the bands.

The IR spectra of all the hBN samples are characterized through two common strong features at 1380 cm^−1^ and 780 cm^−1^, assigned to the conventional BN in-plane and out-of-plane vibrations, and to the signal due to the B-H stretching vibration, at 2450 cm^−1^. These bands are typical of boron nitride samples [58,59].

In the spectra of the hBN-OH_as_ (Figure 3) and hBN-OH_w_ (Figure 4), new weak absorptions are detectable, at 3470 cm^−1^ and at 3250 cm^−1^, for the samples milled for 5 (b) and 10 (c) h. They were attributed to N-H stretching and to O-H stretching, respectively. The intensity of the bands increases with the milling time. It can also be observed that the relative intensity of the bands assigned to the OH stretching and to the NH stretching is different in the hBN-OH_as_ (Figure 3) and hBN-OH_w_ (Figure 4) samples. The washed samples show a greater intensity of the -OH bands: this is clear through a comparison of the bands before and after the treatment and after the normalization of all spectra on the intensity of the band due to the B-N in-plane signal at 1380 cm^−1^.

*Thermogravimetric analysis:* TGA analyses were performed on the hBN samples milled for 5 h and 10 h, in the 30–900 °C temperature range, with a heating rate of 10 °C/min under air. Data of mass losses, obtained from TGA analysis, are in Table 4. The thermographs are shown in Figure S2 in the Supplementary Materials.

A three-step decomposition profile can be observed. The first mass loss below 150 °C can be attributed to the loss of a low molecular mass substance: in this case, water. The second and third step, between 150 °C and 900 °C, can be attributed to defects in the polycyclic system and to the decomposition of nitrogen- and oxygen-containing functional groups. The residue at temperatures higher than 900 °C is due to the inorganic hBN. The amount of mass loss in the 150–900 °C temperature range was found to increase with the ball-milling time, as should be expected; the mechanical treatment creates defective structures which are high-energy sites—these are suitable for functionalization. By assuming a high reactivity of the defective sites, the mass loss in the 150–900 °C range could be tentatively attributed to the thermal degradation of the functional groups. A greater amount of the mass loss in the 150–900 °C temperature range is accompanied by a greater amount of mass loss at T < 150 °C, which was attributed to water. By taking the whole amount of mass loss in the 150–900 °C temperature range, there is not an appreciable difference between the hBN-OH_as_ and hBN-OH_w_ samples; this might indicate that the ball-milling treatment could lead to the modification of hBN without the washing treatment. The mass loss was in a range from 3 to 4% for 5 h milling and in a range from 6% to 7% for 10 h milling. However, a different distribution of mass losses, at T < 500 °C and >500 °C, can be observed. Considering this finding in the light of the above-reported IR results, which revealed a greater amount of OH groups in the washed samples, one could assume that the mass loss in the 150–500 °C temperature range is prevailingly due to OH, as the functional groups. However, further investigations are needed to elucidate this point. 

**Figure 5:**
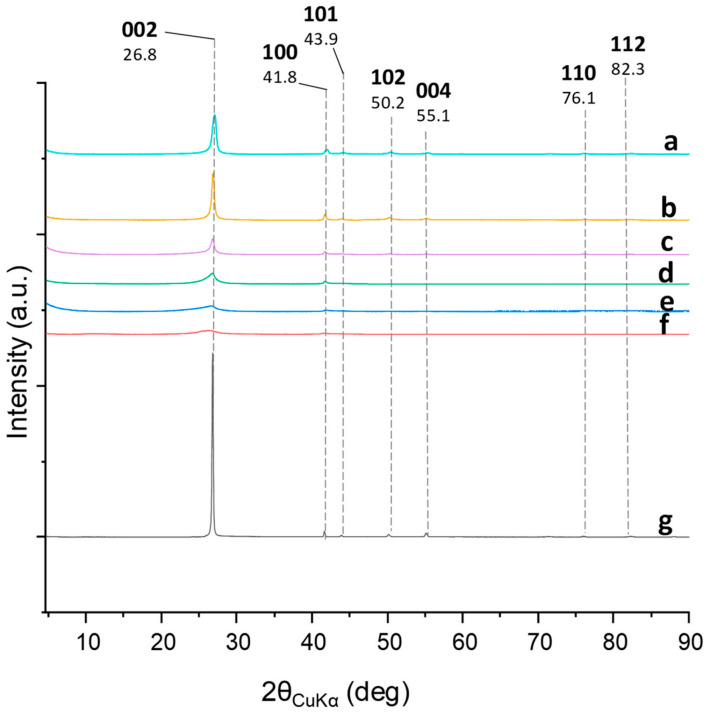
WAXD patterns: hBN-OH_as_, milled for 2.5 (a), 5 (c), and 10 (e) h; hBN-OH_w_, milled for 2.5 (b), 5 (d), and 10 (f) h; hBN-p (g).

*WAXD analysis:* Organization at the solid state of the hBN-OH samples, before and after washing, was investigated by performing WAXD analysis. WAXD patterns of the powders of the ball-milled samples which were milled for 2.5, 5, and 10 h are shown in Figure 5. 

In hBN, the crystalline order in the orthogonal direction to the structural layers in the hBN samples is detected through two (00ℓ) reflections: 002 at 26.8° (interlayer distance of 0.310 nm) and 004 at 55.12°. The in-plane order is shown by 100 and 110 reflections, at 41.8° and 76.1°, respectively [60].

The diffraction peaks at 2θ values of 26.8° (002), 41.8° (100), 43.91° (101), 50.20° (102), 55.12° (004), 76.1° (110), and 82.30° (112) were observed in all the patterns. The interlayer distance, the dimension of the crystallites, and the number of stacked layers were calculated by applying the Bragg’s Law and the Scherrer equation, as explained in the experimental part. In brief, the perpendicular length of the crystallite (D_⊥_) was obtained using the (002) reflection, while the in-plane length (D*_//_*) was determined from the (100) reflection. The dimensional anisotropic factor of the material is given by the relation D*_//_*/D_⊥_. Data are given in Table 5.

The (002) reflection was found in all the spectra and indications of intercalation of low-molar-mass chemicals do not arise from the analysis of the spectra. This could lead to the assumption that the functionalization occurred in peripheral positions, mainly on the edges. 

The milling led to a reduction in the dimension of the crystallites in an orthogonal direction to the structural layers. Similar values were obtained after 2.5 and 5 h milling and a further remarkable reduction was observed after 10 h milling. As a consequence, the number of stacked layers was reduced. The sample milled for 10 h was indeed a few layers hBN, with a number of stacked layers lower than 10. Washing with water appears to promote a minor re-stacking. This can be explained with the adopted procedure, particularly the filtration, which enables the parallel placement and the overlapping of the layers.

The milling also led to a reduction in the dimension of the crystallites inside the basal plane, particularly after 10 h milling. 

**Figure 6:**
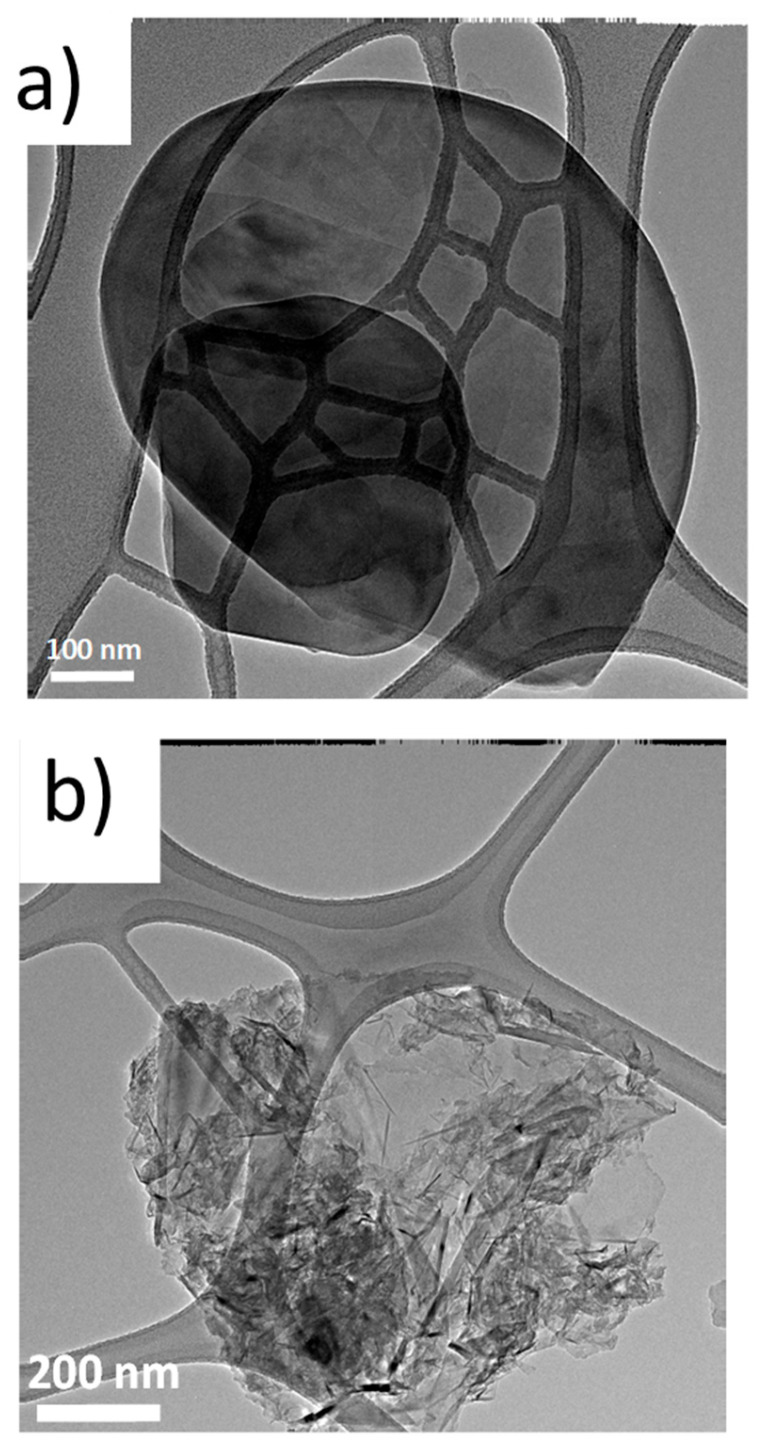
Micrographs of pristine hBN (**a**) and hBN-OH_as5h_ (**b**).

*Transmission electron microscopy*: The morphology of the hBN samples was studied through HR-TEM. Water suspensions of pristine hBN and exfoliated hBN samples (hBN-OH), with 1 mg/1 mL as the concentration, were prepared and poured on the grid. Micrographs were taken at lower and higher magnification levels and are shown in Figure 6: hBN-p in Figure 6a and hBN-OH_as_ in Figure 6b. 

In the low-magnification image of thBNp, the hBN layers appear to have more similar dimensions; however, the lateral sizes for both hBN-p and hBN-OH_as_ appear to be similar, in the range from 500 to 700 nm.

#### Scanning Electron Microscopy Coupled with Energy-Dispersive X-ray Spectroscopy (SEM-EDS)

The morphology of hBN samples was further investigated through SEM-EDS. The powders of both hBN-p and hBN-OH_w10h_ samples were laid on aluminum stubs with the use of a conductive carbon bioadhesive. The samples were then coated in gold by a sputter coater and analyzed.

**Figure 7:**
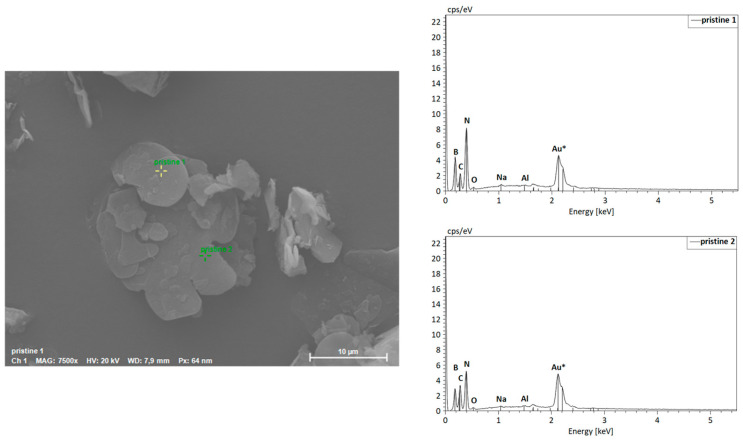
SEM image and EDS spectra of hBN-p.

**Figure 8:**
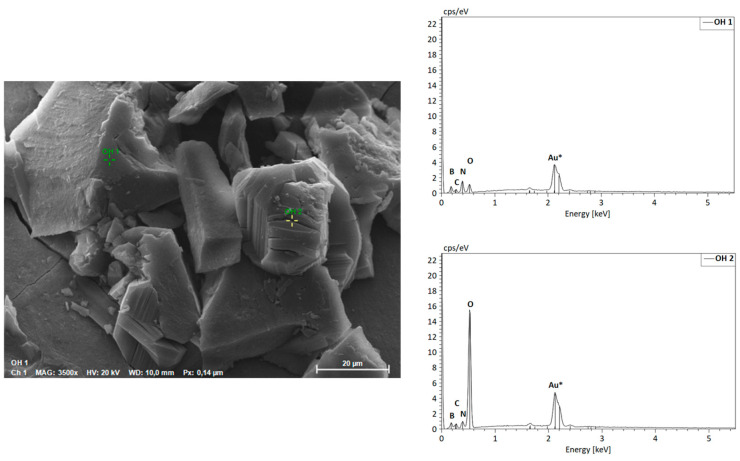
SEM image and EDS spectra of hBN-OH_w10h_.

**Figure 9:**
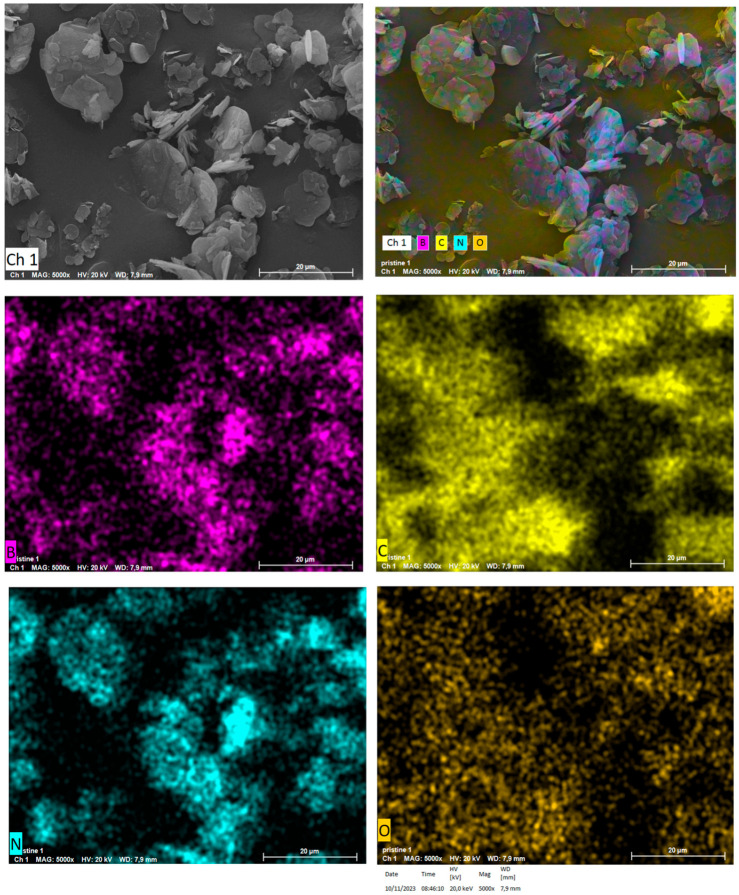
SEM colored image and elemental imaging of hBN pristine.

**Figure 10:**
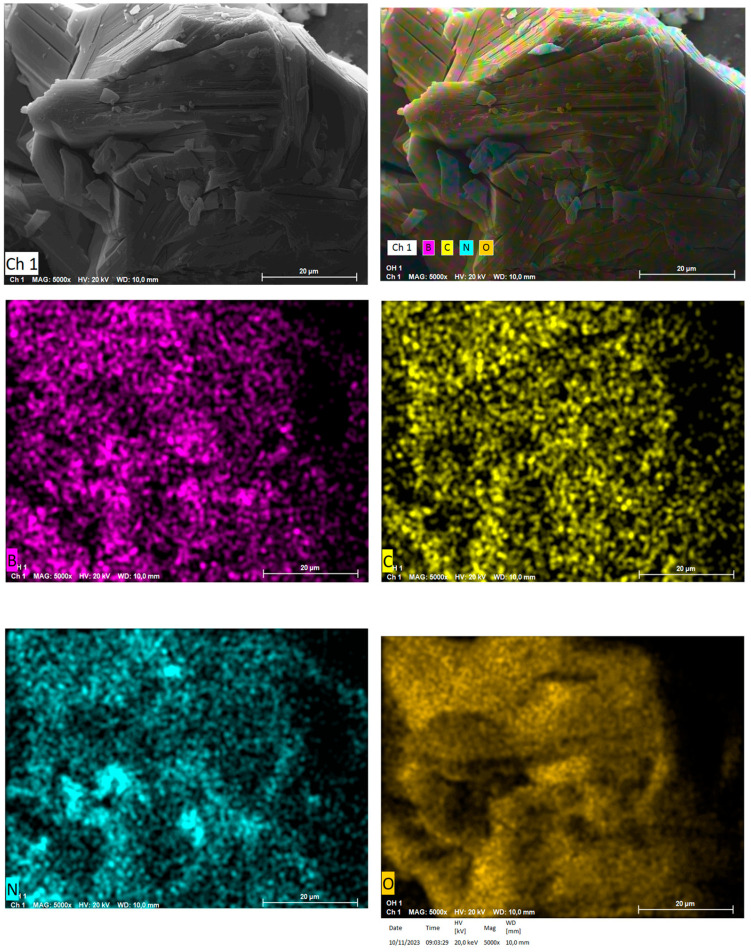
SEM colored image and elemental imaging of hBN-OH_w10h_.

The elemental spectroscopy was evaluated at different points, as shown in Figure 7 for hBN-p and Figure 8 for hBN-OH_w10h_. The EDS maps for hBN-p and hBN-OH_w10h_ are shown in Figure 9 and Figure 10, respectively.

The morphology of the sample appears to be modified, as hBN-p results in a well-distributed flake-like appearance, while hBN-OH_w10h_ is shown to have a very coarse and compact structure.

The EDS maps and spectra, however, confirm the increased presence of oxygen in the hBN-OH_w10h_ sample with respect to the pristine powder. This result confirms the FTIR evidence of increased intensities of bands related to OH groups.

### 3.3. On the Functionalization of hBN with OH Groups

The experimental results reported above reveal that the milling treatment led to hBN samples with lower thermal stability, with the presence of -NH- and -OH functional groups. It can be reasonably commented that the mechanical treatment generates high-energy sites, which are able to react with the molecules present in the headspace of the jar. The formation of amino-functional groups may be a result of a reduction process. The amount of OH groups was found to increase with the milling time (see spectra b and c in Figure 3) and by washing the samples with H_2_O. The OH groups could be due to the reaction of the weak nucleophile/base H_2_O with boron, which acts as an acid in a Lewis acid-base reaction. An extensive milling (10 h time) led also to a more extended rupture of the hBN structure, as suggested by the remarkable ammonia smell felt at the opening of the jar. The functionalization of hBN achieved in the present work occurred in milder and easier experimental conditions with respect to those reported in the literature [45,55]. 

It is indeed worth emphasizing that both the functional groups promote the interaction of hBN with silica and, in the case of OH, could also form a chemical bond. These comments must be considered as working hypotheses. Research is in progress to elucidate the mechanism of the hBN functionalization. The results of this will be reported in a future manuscript. 

### 3.4. Elastomer Composites Containing Silica, hBN-p, and hBN-OH 

Elastomer composites were prepared, with S-SBR and NR as the rubber matrix and silica as the nanostructured filler. The reason for selecting S-SBR was the high vinyl content of the butadiene fraction, which allows a high reactivity with the sulfur-based crosslinking system and silane TESPT, and the coupling agent of silica with the elastomer chains. The combination of S-SBR and NR is suitable for tire tread composites. A minor amount of silica was replaced with either hBN-p or hBN-OH: 15% and 30% by volume, maintaining the same total volume of the filler. A sample of hBN-OH was selected: the one milled for 5 h and then washed. It will be indicated as hBN-OH in the text below. As shown above by FT-IR and TGA analyses, 5 h milling allowed the introduction of a low yet appreciable amount of OH groups and did not remarkably alter the structure of the hBN layers, which maintained an order in the basal planes and lateral sizes that were similar to the one of pristine hBN. The sulfur-based crosslinking and the static and dynamic mechanical properties are discussed next.

#### 3.4.1. Sulfur-Based Crosslinking

Sulfur-based crosslinking was performed at 170 °C for 10 min in a rubber process analyzer, as described in detail in the Experimental Section, obtaining torque vs. time curves. The minimum torque (M_L_), the maximum torque (M_H_), the time required to have a torque equal to M_L_ + 1 (t_S1_), i.e., the induction time of crosslinking, and the time required to reach 90% of the maximum torque (t_90_), i.e., the optimum time of crosslinking, were measured. The data are shown in Table 6. The corresponding curves are shown in Figure S3 in the Supplementary Materials. 

The curing rate was calculated by using the formula in Equation (4).

(4) $$Curing\;rate=\frac{M_{H}-M_{L}}{T_{90}-T_{S1}}$$

M_L_ values are assumed as an index of the viscosity of the composites. The replacement of silica with hBN led to the reduction in M_L_, greater for a greater amount of silica replaced. Remarkably lower M_L_ values were obtained with hBN-p, whereas the difference with the silica-based composites was slight in the case of hBN-OH. In the introduction, it was reported that a 2D nanomaterial such as hBN, which does not have a structure as silica [5], was supposed to interrupt the 3D network of silica and to weaken the supramolecular interactions among the functional groups on the silica surface. It seems that this was achieved particularly with hBN-p, which has a lower compatibility with silica than hBN-OH, which bears edge -NH- and -OH groups. It is worth adding that hBN-OH has a lower number of stacked layers than hBN-p; hence, it can give rise to a more extended interaction with silica. The (M_H_−_L_) values were lower for the composites with hBN and slight differences were obtained in the presence of either hBN-p or hBN-OH. This finding led the researchers to suppose that the M_H_ difference was mainly due to the different extents of the filler networks.

The replacement of silica did not have a remarkable effect on the induction time of crosslinking, and did not have an effect on the optimum time or on the curing rate when hBN-OH was used; in contrast, longer t_90_ and a lower curing rate were obtained with hBN-p. By taking into account the fact that the kinetics of curing are determined through measuring the torque, these findings could be explained by the ability of hBN-OH to interact with silica and to contribute to the formation of a network during the crosslinking reaction.

#### 3.4.2. Dynamic Mechanical Properties in the Shear Mode

**Figure 11:**
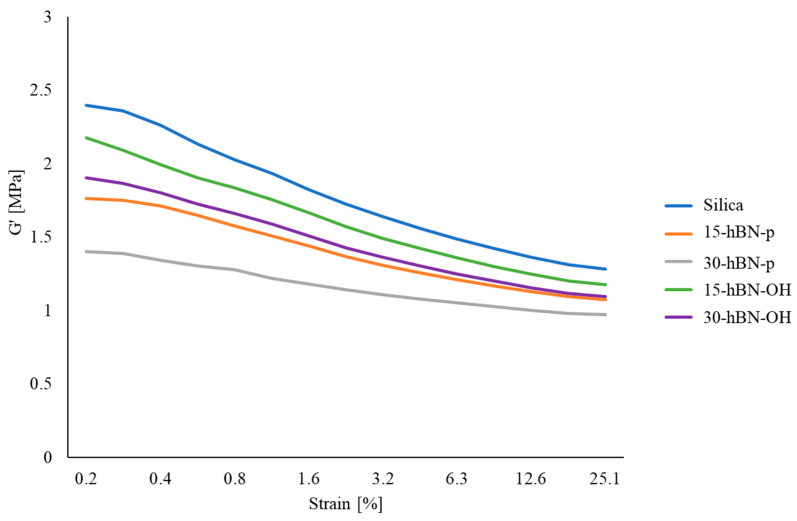
G′ vs. strain for composites of Table 1.

**Figure 12:**
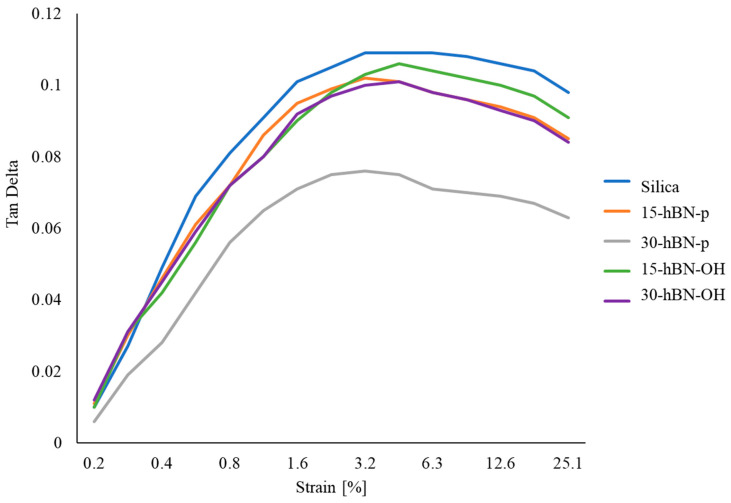
Tan delta vs. strain for composites of Table 1.

Dynamic mechanical properties in the shear mode were determined through strain sweep experiments at 50 °C by following the procedure described in the Experimental Section, with the strain amplitude in the range from 0.2% to 25%. The storage modulus (G′) and the loss modulus (G″) were measured and their ratio G″/G′ (=Tan δ was elaborated. Data of G′_γmin_, G′_γmax,_ ΔG′, ΔG′/G′_γmin_, G″_max_, and tanδ_max_ are presented in Table S1 in the Supplementary Materials. The curves of the G′ vs. strain and the Tan delta vs. strain are shown in Figure 11 and Figure 12, respectively. The curve of the G″ vs. strain and the curve of the G″ vs. G′ are shown in Figure S4 and in Figure S5, respectively, in the Supplementary Materials. 

G′ at minimum strain is an index of the presence of the filler network. A filler network is formed by the interaction of filler particles, either directly or mediated by polymer chains. The reduction in G′ with the strain amplitude, i.e., the nonlinearity of the storage modulus, is known as Payne effect [61,62,63]; this is due to the disruption of the filler network. Parameter $\frac{\Delta G’}{{G’}_{\gamma min}}$ is a normalized index of the Payne effect. 

The replacement of silica with hBN, both pristine and functionalized, had effects—which consistently increased with the extent of the replacement—on the low-strain dynamic mechanical properties of the composites. 

hBN-p in place of silica led to remarkably lower values of G′_γmin_, G′_γmax,_, ΔG′/G′_γmin_, G″_max_, and Tan delta. These findings arise from a lower extent of the filler network and indicate a lower Payne effect and a lower dissipation of energy. They suggest the powerful effect of hBN-p for disrupting and preventing the formation of a silica-based filler network. However, to account for these results, the lamellar structure of hBN and the procedure used for preparing the specimen to be analyzed—shown in Figure S1 in the Supplementary Materials—should be taken into account. The uncured composites, taken from the internal mixer, were processed on a two-roll mill and were then placed in the rheometer. The hBN layers are reasonably arranged parallel to the plate of the rheometer. In this configuration, the layered 2D nanofiller can express its lubricant effect, which is well documented in the literature [64,65,66]. This behavior has also been reported for nanosized graphite nanoplatelets (GnP) [43]. The anisotropy of nanocomposites based on 1D and 2D nanofillers, such as GnP and carbon nanotubes, has been documented in the literature [67,68]. 

With hBN-OH in place of silica, analogous effects were observed. However, with respect to hBN-p, higher values of G′ were observed at both the minimum and the maximum strain; higher values of G′_γmax_, ΔG′/G′_γmin_, G″_max_, and Tan delta were also observed here. These findings appear to be in line with the observations from the crosslinking experiments: hBN-OH has a better interaction with silica; the extent of the filler network is lower with respect to the composite, with silica as the only filler, but it is larger than that in the presence of hBN-p. Moreover, the interaction with silica could hinder, to some extent, the parallel placement of the layers commented on above. 

#### 3.4.3. Dynamic Mechanical Properties from Axial Compression Tests

**Figure 13:**
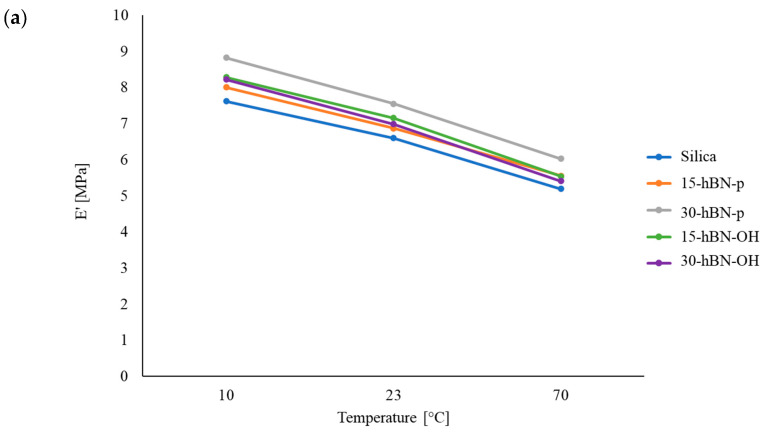

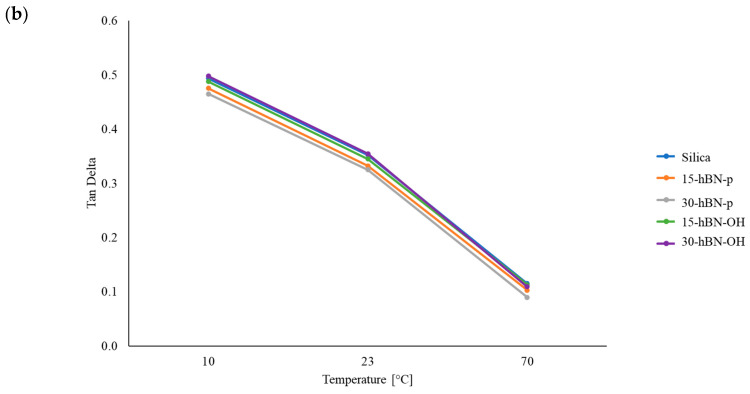
Axial dynamic mechanical properties of S-SBR 4630 compounds measured in compression: (**a**) storage modulus vs. temperature; (**b**) tan delta vs. temperature.

Dynamic mechanical properties were determined in the axial mode by applying the sinusoidal stress in compression, with a pre-strain of −25%, to remove the filler network or—at least—to reduce it significantly. Details of the procedure are provided in the Experimental Section. Data of E′, E″, and Tan δ, measured at 10 °C, 23 °C, and 70 °C, respectively, are provided in Table 7; the dependences of E′ and Tan δ on temperature are shown in Figure 13a and Figure 13b, respectively. The dependence of E″ on the temperature is shown in Figure S6 in the Supplementary Materials. 

An increase in E′ was obtained at all the temperatures, with both hBN-p and hBN-OH in the place of silica. The values of E’ increased with the hBN-p content and the difference with respect to the silica composite was about 15% for 30% replacement at 70 °C. On the contrary, the E’ values were very similar in both the composites with hBN-OH. These results could be—to some extent—unexpected. In fact, it was mentioned above that hBN-p should have a poor interaction with silica, whereas hBN-OH should establish interactions and even chemical bonds through the functional groups. However, these data could be explained by taking the procedure into consideration (procedure shown in Figure S1 in the Supplementary Materials); this procedure was used for the preparation of the specimens to be analyzed. As explained above, the uncured composite from the mixer was processed on the two-roll mill. To measure the axial dynamic mechanical properties, small cylinders were then prepared by rolling up the rubber sheet obtained from the two-roll mill. The applied sinusoidal stress was found to be parallel to the longest direction of the cylinder. Platelets lay along such a direction and experienced the applied stress. The increase in the dynamic rigidity measured in the axial mode, in the presence of the reduction in the shear moduli, could thus be explained with the anisotropy of the nanocomposites [65,66]. The results obtained with hBN-OH could be explained better with the more successful hBN-OH/silica interaction, which could also lead to a less parallel orientation of the hBN-OH layers. Dwelling upon the tan delta values of the composites with hBN-OH, they are in line or even higher at low temperatures and lower at high temperatures. This is an indication of the interaction between the filler network formed by silica and hBN-OH and the polymer chains, through the sulfur-based silane (TESPT), which acts as coupling agent.

#### 3.4.4. Tensile Properties

**Figure 14:**
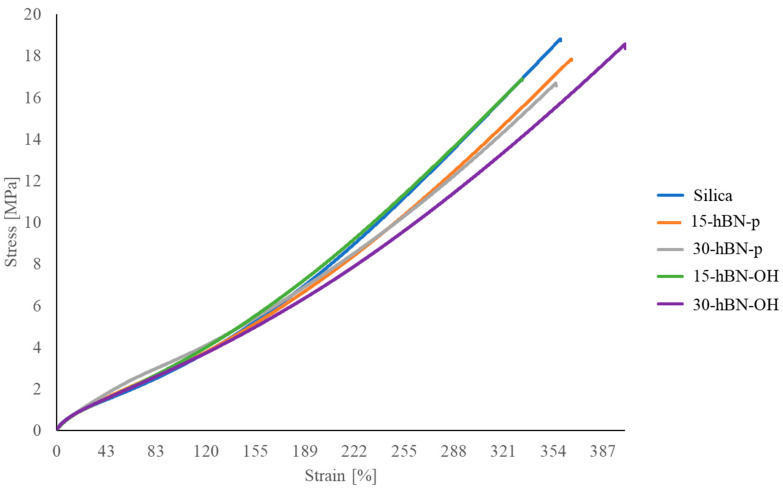
Tensile curves of S-SBR 4630 compounds obtained through stress–strain experiments.

Tensile properties were measured at a nominal room temperature through quasi-static measurements. The values of the stresses at different elongations, stresses, and energies at break are shown in Table 8; the curves of stress vs. strain are shown in Figure 14.

The partial replacement of silica with hBN, either pristine or functionalized, brought about an increase in the stresses at low elongations, up to 100%. In the case of hBN-p, the values at 300% elongation and the ultimate properties are substantially in line with those of the reference composite for 15% replacement, whereas they are lower in the presence of a greater amount of hBN. These findings, in particular the higher rigidity at low strains, are typical of a nanofiller with high aspect ratio and have been observed also in the case of nanofillers such as carbon nanotubes and sepiolite [68,69]. Similar behavior is shown by 15-hBN-OH, whereas the composite with 30% hBN shows better ultimate properties. Without stretching too far into making comments that are not supported by experimental data, one could hypothesize a positive role played by the tight interaction of the silica/hBN-OH system with the elastomer chains. 

#### 3.4.5. High-Pressure Capillary Viscometer (HKV)

**Figure 15:**
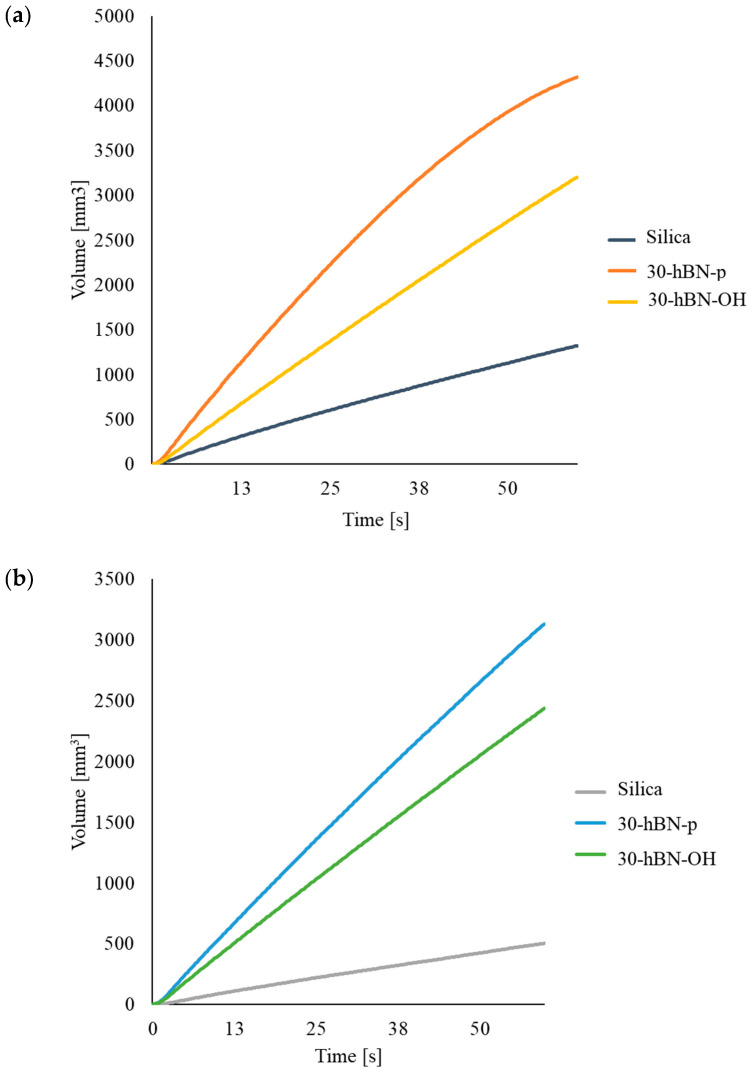
Measurement in mm^3^ of composites released by the high-pressure capillary viscometer vs. time: (**a**) immediately after compounding, (t = 0); (**b**) after 7 days (t = 7).

The poor rheological properties of silica-based composites were mentioned in the Introduction. The investigation of the rheological properties of the composites shown Table 1 (i.e., the investigation of the effect of hBN, both pristine and functionalized) was performed through high-pressure capillary viscometer (HKV) tests; the methods of these are described in Section 2 The experiments were carried out on the composite with silica as the only filler and on composites with a replacement of 30% in volume of silica, with hBN-p and hBN-OH. Data were collected immediately after the compounding, at t = 0, and after 7 days of storage. Results are reported in Figure 15, Figure 15a, and Figure 15b, respectively. In the graphs, the volume (mm^3^) of composites released by the instrument was plotted versus time.

At t = 0, the 30% hBN-p-based composite exhibits the best processability: the largest volume of material flows per unit of time. The lowest amount of extruded composite was obtained with the composite with only silica as the filler, whereas the 30% hBN-OH compound had an intermediate behavior.

After 7 days storage, the curves of the different composites showed the same relative placement, and those of the hBN-p- and hBN-OH-based compounds appear closer to each other. The two composites with hBN show remarkably better processability in comparison with the composite with only silica as the filler: the amount of composite extruded from the viscometer, with respect to the silica-based composite, was about 500% more with hBN-p and about 400% more with hBN-OH.

Hence, hBN-p and hBN-OH provide substantially improved rheological behavior of the silica-based elastomer composite, both immediately after compounding and after 7 days of storage. The much better processability after 7 days indicates that the shelf life of the silica-based composites can be improved by adding either hBN-p or hBN-OH. These findings are reasonably a consequence of the lower extent of the filler network. The better results obtained with hBN-p could be explained with the poorer interaction with silica.

#### 3.4.6. Transmission Electron Microscopy Analyses (TEM) of Rubber Composites

**Figure 16:**
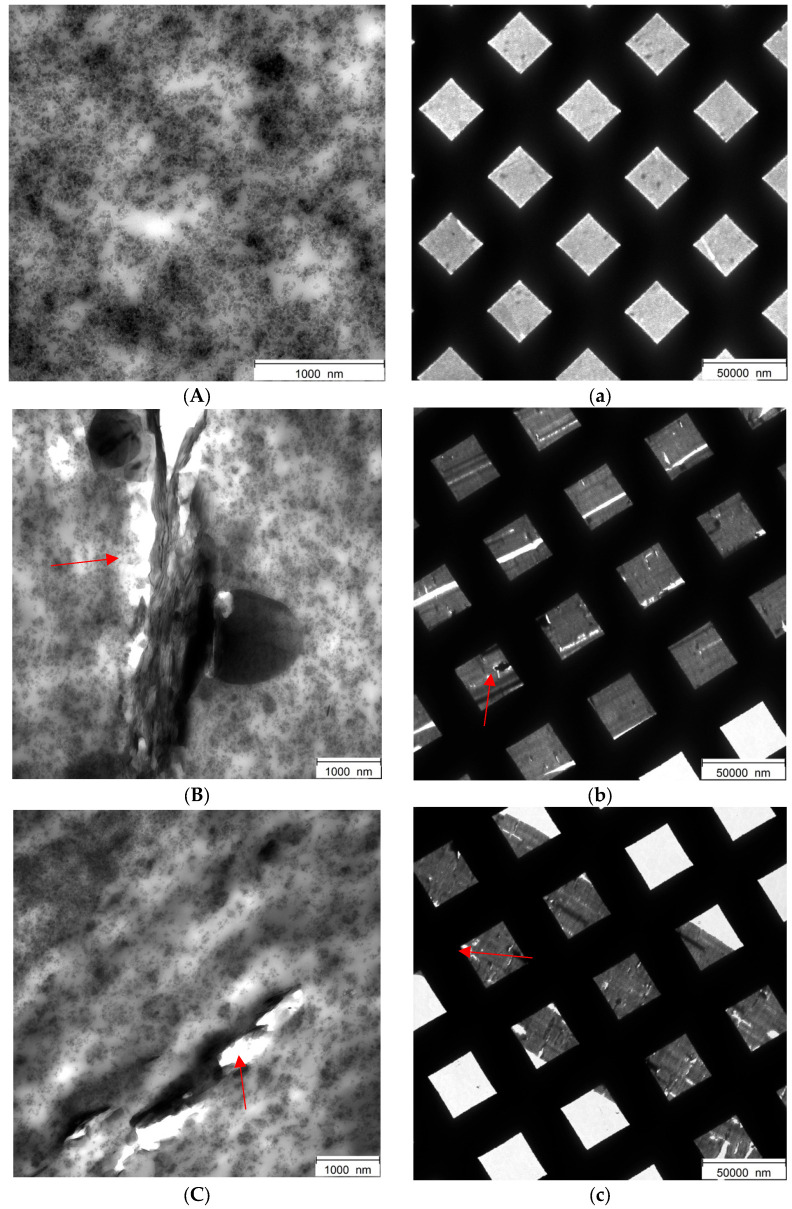

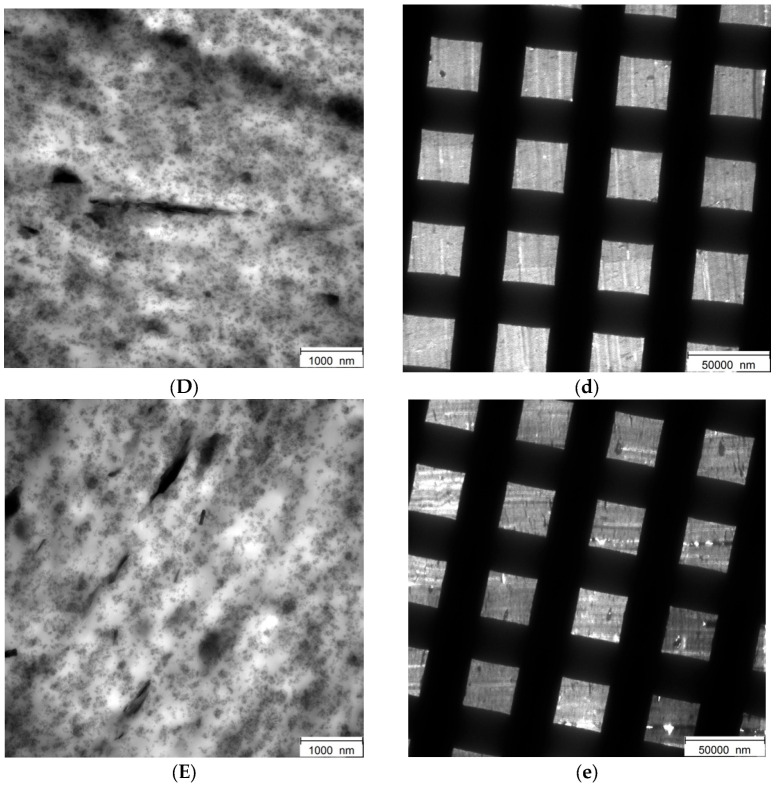
Micrographs of the following composites: silica (**A**,**a**), 15hBN-p (**B**,**b**), 30-hBN-p (**C**,**c**), 15-hBN-OH (**D**,**d**), 30-hBN-OH (**E**,**e**), at higher magnifications (**A**–**E**) and at lower magnifications (**a**–**e**).

Transmission electron microscopy (TEM) analysis was performed with the aim of investigating the filler dispersion on the vulcanized composites of Table 1, hence with silica as the only filler and with the silica/hBN-p and silica/hBN-OH as the hybrid filler systems. TEM micrographs are shown in Figure 16. Fifteen low-magnification micrographs and fifteen high-magnification micrographs were taken for each composite; representative images were selected. Low-magnification micrographs, where the TEM grid is visible, were taken to allow the observation of a larger area of sample, thus making a first evaluation of filler dispersion possible, although these are still related to a restricted sample area. Red arrows highlight sample flaws in the selected micrographs.

The micrographs of the composites with 15% silica replacement are shown in Figure 16b (hBN-p) and Figure 16d (hBN-OH); those of the composites with 30% silica replacement are shown in Figure 16c (hBN-p) and Figure 16e (hBN-OH). 

Micrographs at high magnification of composites with hBN-p reveal the presence of flaws; more specifically, they show detachments of the elastomer matrix from the filler, at both levels of hBN-p content: 15% (Figure 16B) and 30% (Figure 16C). On the contrary, no large flaws are visible in the high-magnification micrographs of the composites with hBN-OH, both at 15% (Figure 16D) and 30% (Figure 16E) content. 

The low-magnification micrograph of the 15% hBN-OH composite (Figure 16d) shows a level of homogeneity that is very close to the one of the reference silica composite (Figure 16a). The low-magnification micrograph of the 30% hBN-OH composite (Figure 16e) highlights a good sample homogeneity, with the presence of a few aggregates.

In general, samples containing hBN-p are characterized by micrographs with large agglomerates that induce points of detachment between the filler and the rubber matrix. Such large agglomerates, with a lamellar structure, can be attributed to hBN and led the researchers to hypothesize the presence of poor hBN-p dispersion. When hBN-OH is employed, a higher homogeneity is observable in both the low- and high-magnification micrographs. A limited number of hBN aggregates is visible with little or no rubber–filler detachments. 

Overall, hBN-OH appears more homogeneously dispersed in the TEM micrographs, leading the researchers to hypothesize the presence of a generally higher level of dispersion of hBN-OH with respect to hBN-p in the considered elastomer composites, at both levels of silica replacement. The best filler dispersion in the presence of hBN seems to be achieved with a 15% replacement of silica with hBN-OH.

The OH groups in hBN-OH appear to favor the compatibilization and the dispersion of hBN-OH. The chemical modification of h-BN could promote the interaction with silica and with silica’s coupling agent, the sulfur-based silane, either directly or mediated by silica. Thus, alongside an improved interaction with silica, the interaction with the rubber matrix could be favored.

## 4. Conclusions

This work demonstrates that a 2D nanomaterial such as hBN, which has attracted enormous interest over recent years for numerous applications in various fields, can also substantially improve the properties of a silica-based elastomer composite when used in the place of a minor amount of silica. hBN-p was a commercially available type and was exfoliated and functionalized using an ecofriendly mechanical treatment—ball milling—without any chemical substance. -NH- and -OH functional groups were introduced on hBN, as shown in the FT-IR spectra. The defective sites created by the milling were able to react with the molecules present in the headspace of the jar as well as with the water used for washing the samples. TGA revealed the lower thermal stability of the milled samples and the maximum number of functional groups was estimated to be about 6% by mass. The functional groups were in the peripheral positions, mainly on the edges, as suggested by the by X-ray analyses, which revealed the persistence of the (002) reflection and the absence of (00l) reflections at lower 2theta angles. The mechanical treatment allowed the researchers to prepare a few layers of hBN-OH. With 5 h as the milling time, the lateral size of the layers was substantially unaltered with respect to hBN-p, as revealed through HR-TEM, and the order in the basal planes was still detected, as shown by WAXD. 

Silica-based elastomer composites were prepared with both hBN-p and hBN-OH, milled for 5 h, in place of 15% and 30% by volume of silica. The processability of the composite was drastically improved, particularly after one week storage at room temperature. The composite extruded using a high-pressure capillary viscometer was increased, with respect to the reference silica composite, by about 500% and 400% using hBN-p and hBN-OH, respectively. Both hBNs, used in the place of silica, promoted a lower Payne effect, a higher dynamic rigidity and similar or lower hysteresis. The results suggest that hBN-p has a more significant ability to disrupt the silica-based network. However, homogenous composites without evidence of flaws in HR-TEM micrographs and with the best dependence on temperature of tan delta were obtained with h-BN-OH, which appears to be the best candidate for the development of elastomer composites for use on a very large scale, such as in tire tread compounds. 

The simple and ecofriendly preparation of hBN-OH appears to be an appropriate solution for scaling up the whole technology.

## Figures and Tables

**Table 1:** Recipes of elastomer composites.

Recipes in phr	Silica	15-hBN-p	30-hBN-p	15-hBN-OH	30-hBN-OH
	[phr]	[phr]	[phr]	[phr]	[phr]
S-SBR 4630	70	70	70	70	70
NR	30	30	30	30	30
Silica	50	42.5	35	42.5	35
hBN	0	7.5	15	0	0
BN-OH	0	0	0	7.5	15

Other ingredients: Silane TESPT 4, stearic acid 2, ZnO 2.5, 6PPD 2, sulfur 2, and TBBS 1.8.

**Table 2:** Values of the wavenumbers corresponding to the absorption peaks of the most intense IR transitions for spectra in Figure 3. The relative abundance (%) is also reported.

Experimental Wavenumber (cm^−1^)	Vibrational Assignment	Relative Abundance per Sample (%)			
		hBN-Oh_as_ Milled for 2.5 h	hBN-Oh_as_ Milled for 5 h	hBN-Oh_as_ Milled for 10 h	hBN-p
3470	N-H stretching	2	3	5	0
3250	O-H stretching	2	3	4	0
2450	B-H stretching	1	1	1	1
1380	BN in-plane	100	100	100	100
780	BN out-of-plane	13	12	9	9

**Table 3:** Values of the wavenumbers corresponding to the absorption peaks of the most intense IR transitions for spectra in Figure 4. The relative abundance (%) is also reported.

Experimental Wavenumber (cm^−1^)	Vibrational Assignment	Relative Abundance per Sample (%)			
		hBN-Oh_w_ Milled for 2.5 h	hBN-Oh_w_ Milled for 5 h	hBN-Oh_w_ Milled for 10 h	hBN-p
3470	N-H stretching	1	2	6	0
3250	O-H stretching	1	2	6	0
2450	B-H stretching	1	1	1	1
1380	BN in-plane	100	100	100	100
780	BN out-of-plane	13	12	9	9

**Table 4:** Mass losses (mass %) for pristine of exfoliated hBN samples before (hBN-OH_as_) and after washing (hBN-OH_w_) from TGA analysis.

Sample	Milling Time (h)	Temperature Range			
		T < 150 °C	150 °C < T < 500 °C	500 °C < T < 900 °C	T > 900 °C + Residue
hBN-p	=	0	0	1.5	98.5
hBN-OH_as_	5	1.9	2.7	1.3	96.5
hBN-OH_w_	5	2.5	2.52	1	93.5
hBN-OH_as_	10	6.8	4.5	1.6	87.2
hBN-OH_w_	10	6.7	6.4	0.8	86.1

The amounts of functional groups were estimated on the basis of the mass loss in the 150 °C–900 °C T range.

**Table 5:** Structural parameters derived from Bragg’s Law and Scherrer equation ^a^.

Sample	Milling Time (h)	d_002_ (nm)	D_⊥_ (nm)	D*_//_* (nm)	D*_//_*/D_⊥_	Layers
hBN-p		0.31	41.53	44.56	1.07	132
hBN-OH_as_	2.5	0.31	12.82	16.80	1.31	41
hBN-OH_w_	2.5	0.31	18.81	26.22	1.39	60
hBN-OH_as_	5	0.31	11.27	19.01	1.68	36
hBN-OH_w_	5	0.32	15.01	21.61	2.32	40
hBN-OH_as_	10	0.32	2.61	3.11	1.19	8
hBN-OH_w_	10	0.32	2.90	3.27	1.13	9

^a^ Equations (1)–(3) are reported in Section 2.

**Table 6:** Vulcanization data of S-SBR 4630-based compounds.

	Silica	15-hBN-p	30-hBN-p	15-hBN-OH	30-hBN-OH
M_L_ [dNm]	3.8	2.5	1.9	3.4	3.1
M_H_ [dNm]	21.1	18.1	16.5	19.4	18.6
M_H_-M_L_ [dNm]	17.3	15.5	14.5	16.0	15.5
t_S1_ [min]	3.1	3.4	3.4	3.2	3.4
t_90_ [min]	7.8	8.5	8.2	7.5	7.7
curing rate [dNM/min]	3.7	3.1	3.1	3.8	3.7

**Table 7:** Axial dynamic mechanical properties of S-SBR 4630 compounds measured in compression.

	T [°C]	Silica	15-hBN-p	30-hBN-p	15-hBN-OH	30-hBN-OH
E′ [MPa]	10	7.6	8.0	8.8	8.3	8.2
	23	6.6	6.9	7.5	7.1	7.0
	70	5.2	5.6	6.0	5.5	5.4
E″ [MPa]	10	3.8	3.8	4.1	4.0	4.1
	23	2.3	2.3	2.4	2.5	2.5
	70	0.6	0.6	0.5	0.6	0.6
Tanδ	10	0.49	0.48	0.47	0.49	0.50
	23	0.35	0.33	0.33	0.35	0.35
	70	0.12	0.10	0.09	0.11	0.11
ΔE′ (E′@10 °C–E′@70 °C) (MPa)		2.4	2.4	2.8	2.7	2.8

**Table 8:** Tensile properties of composites of Table 1.

	Silica	15-hBN-p	30-hBN-p	15-hBN-OH	30-hBN-OH
σ_50%_ [MPa]	1.6 ± 0.02	1.8 ± 0.03	2.1 ± 0.05	1.7 ± 0.03	1.7 ± 0.013
σ_100%_ [MPa]	2.9 ± 0.01	3.2 ± 0.06	3.5 ± 0.1	3.3 ± 0.07	3.1 ± 0.05
σ_300%_ [MPa]	7.9 ± 0.21	7.5 ± 0.13	7.6 ± 0.36	8.4 ± 0.34	6.9 ± 0.23
σ_break_ [MPa]	18.7 ± 0.65	17.8 ± 1.59	16.6 ± 1.61	16.8 ± 2.60	18.4 ± 1.28
Ɛ_break_ [%]	361.3 ± 9.62	359.1 ± 23.24	343.2 ± 20.24	325.9 ± 22.86	401.9 ± 21.67
Energy break [MJ/m³]	28.2 ± 1.80	26.8 ± 3.54	24.4 ± 3.54	23.2 ± 3.84	31.5 ± 3.38

## Data Availability

Data is contained within the article.

## Supplementary Materials

The following supporting information can be downloaded at: https://www.mdpi.com/article/10.3390/nano14010030/s1, Figure S1: Procedures for the preparation of specimens for axial dynamic-mechanical analyses (a) and shear dynamic mechanical analyses (b); Figure S2: TGA thermograms under air of hBN-OHas5h (a); hBN-OHw5h (b); hBN-OHas10h (c) and hBN-OHw10h; (d). Pristine hBN (e); Figure S3: Crosslinking curves of composites of Table 1; Table S1: Shear dynamic-mechanical properties from strain sweep experiments of composites of Table 1; Figure S4: G″ vs. strain for composites of Table 1; Figure S5: G plot for for composites of Table 1; Figure S6: E″ vs. Temperature for for composites of Table 1.
