# Supplementary material for: Hexagonal Boron Nitride as Filler for Silica-Based Elastomer Nanocomposites
Source: Nanomaterials (Basel). 2023 Dec 21;14(1):30. doi: 10.3390/nano14010030 (PMC10780802; doi:10.3390/nano14010030)
Supplement: Supplementary file 1 [file nanomaterials-14-00030-s001.zip › nanomaterials-2640377-supplementary.pdf]

nanomaterials

Supplementary Materials

Hexagonal Boron Nitride as Filler for Silica-Based Elastomer Nanocomposites

Federica Magaletti [1], Gea Prioglio [1], Ulrich Giese [2], Vincenzina Barbera [1,*] and Maurizio Galimberti [1,*]

[1] Department of Chemistry, Materials and Chemical Engineering "G. Natta", Politecnico di Milano, Via Mancinelli 7, 20131 Milano, Italy; federica.magaletti@polimi.it (F.M.); gea.prioglio@polimi.it (G.P.)
[2] Deutsches Institut für Kautschuktechnologie e. V., Eupener Straße 33, 30519 Hannover, Germany
* Correspondence: vincenzina.barbera@polimi.it (V.B.); maurizio.galimberti@polimi.it (M.G.)

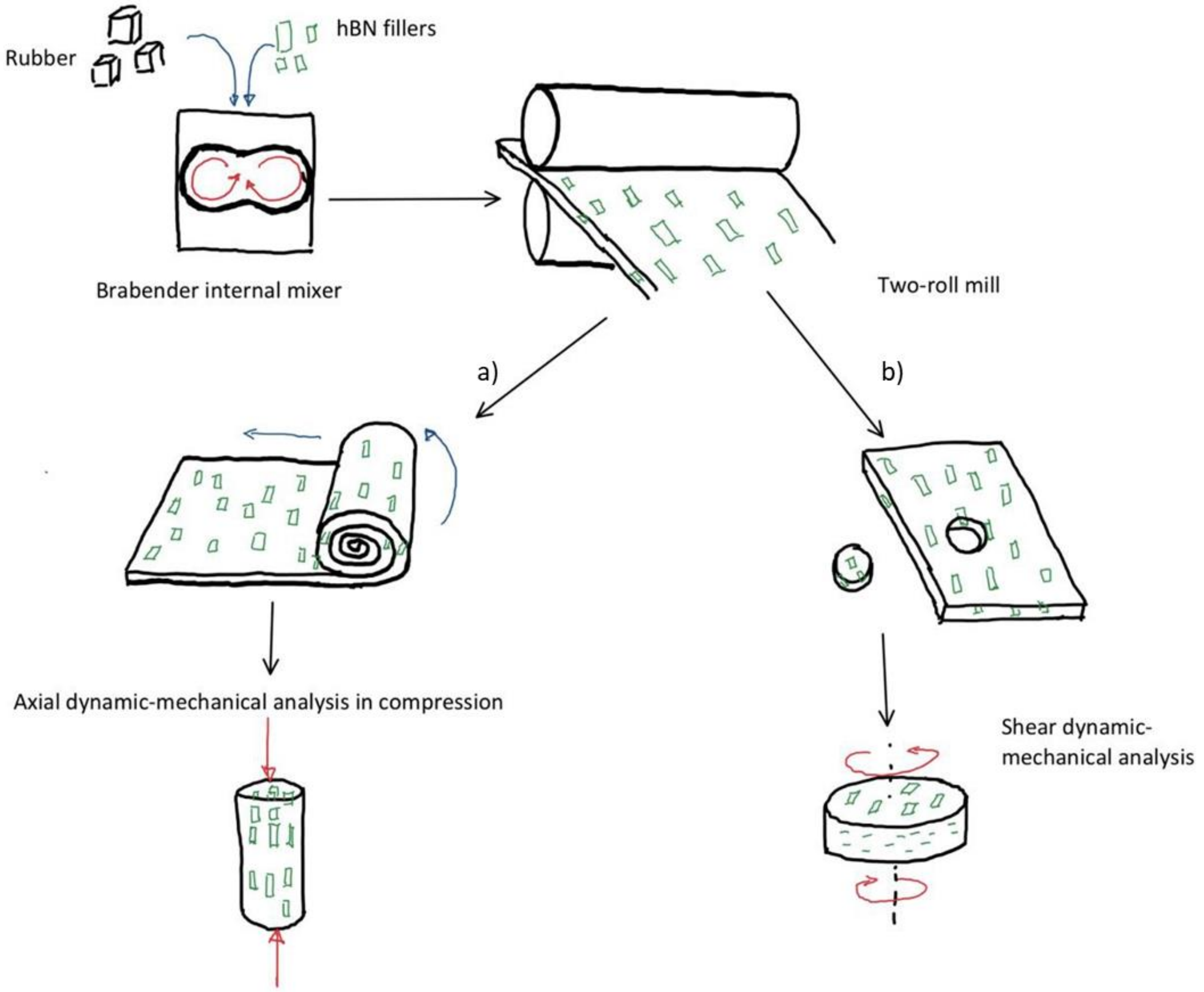

Figure S1. Procedures for the preparation of specimens for axial dynamic-mechanical analyses (a) and shear dynamic mechanical analyses (b).

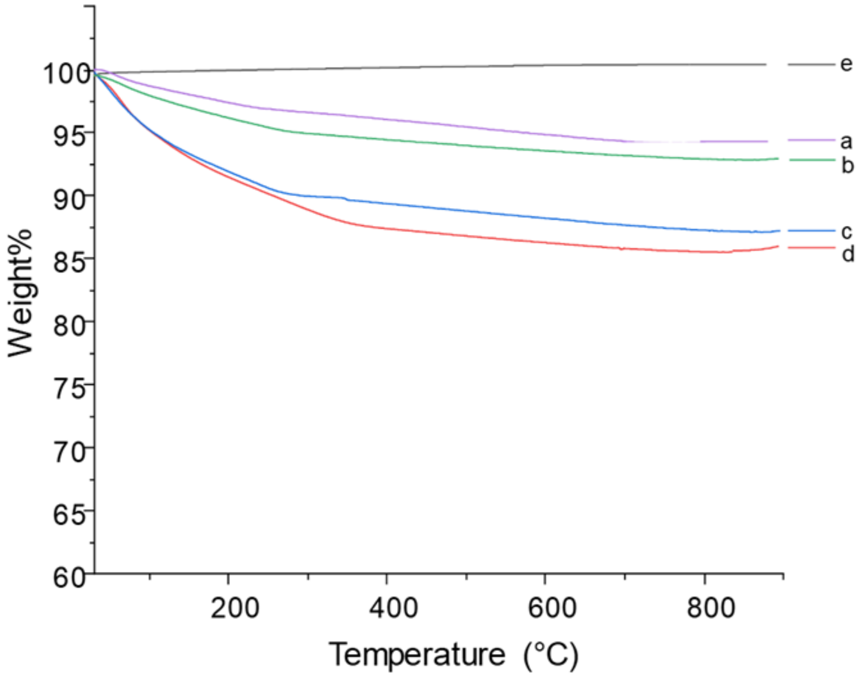

Figure S2. TGA thermograms under air of hBN-OH_{as5h} (a); hBN-OH_{w5h} (b); hBN-OH_{as10h} (c) and hBN-OH_{w10h}; (d). Pristine hBN (e).

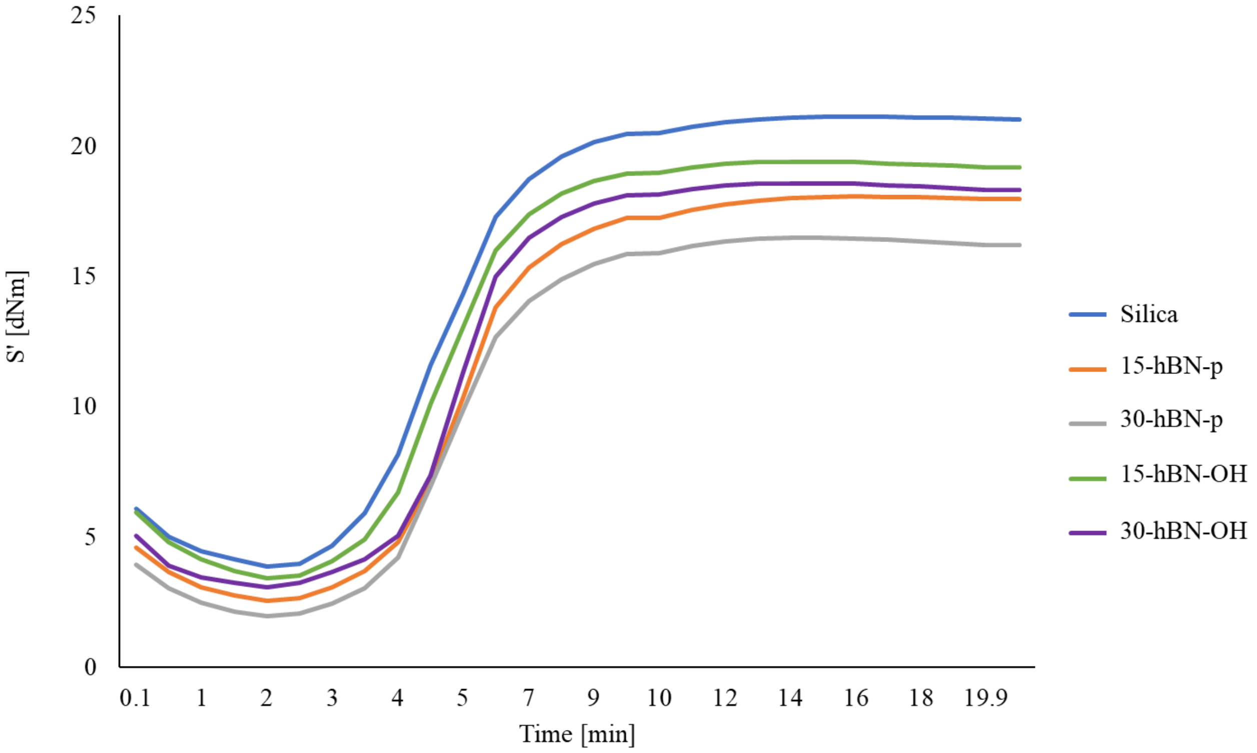

Figure S3. Crosslinking curves of composites of Table 1.

Table S1. Shear dynamic-mechanical properties from strain sweep experiments of composites of Table 1.

	Silica	15-hBN-p	30-hBN-p	15-hBN-OH	30-hBN-OH
**$G'_{\gamma min}$ [Mpa]**	2.40	1.76	1.40	2.17	1.90
**$G'_{\gamma max}$ [MPa]**	1.28	1.08	0.97	1.18	1.09
**$\Delta G'$ [MPa]**	1.11	0.69	0.43	0.99	0.81
**$\Delta G'/G'_{\gamma min}$**	0.46	0.39	0.31	0.46	0.43
**G''_{max} [MPa]**	0.18	0.14	0.09	0.15	0.14
**$\tan\delta_{max}$**	0.11	0.10	0.08	0.11	0.09

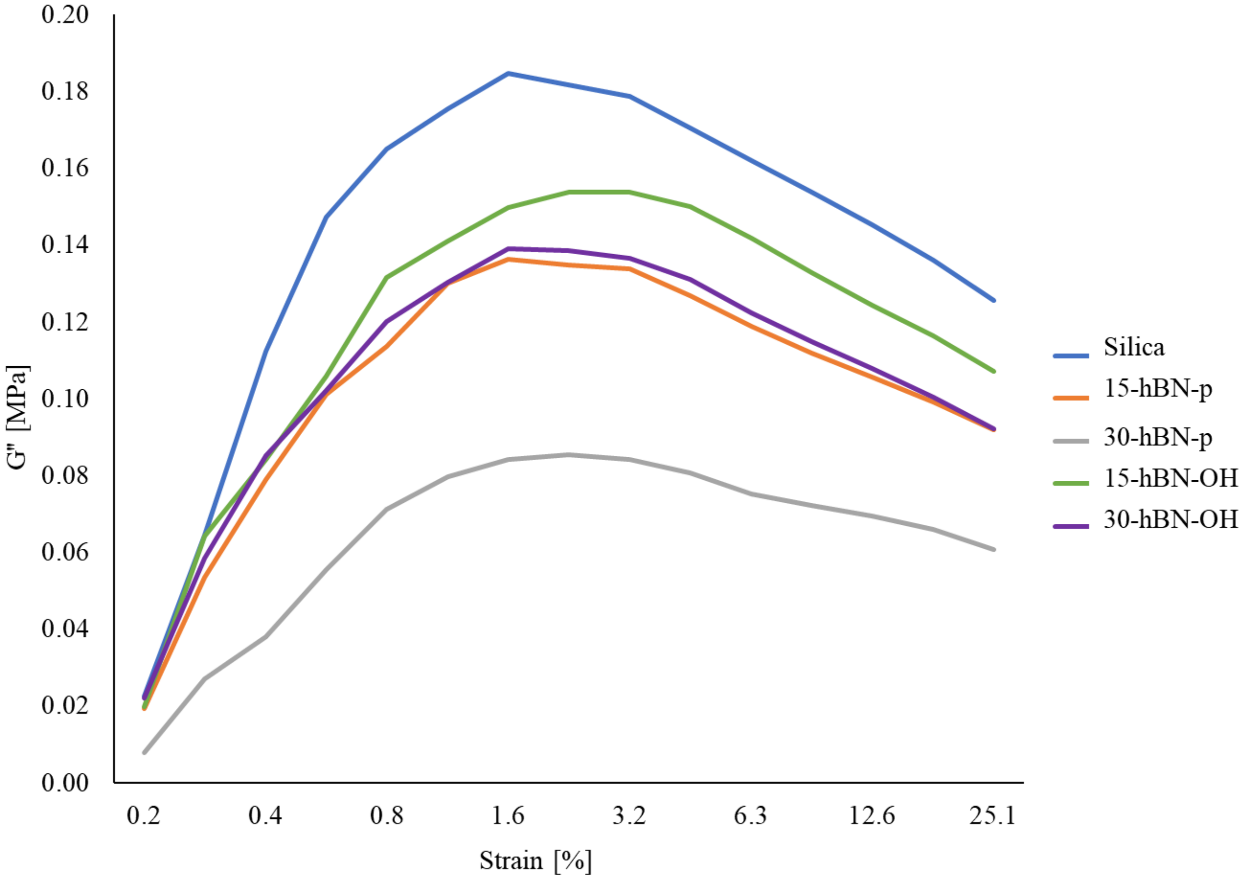

Figure S4. G'' vs strain for composites of Table 1.

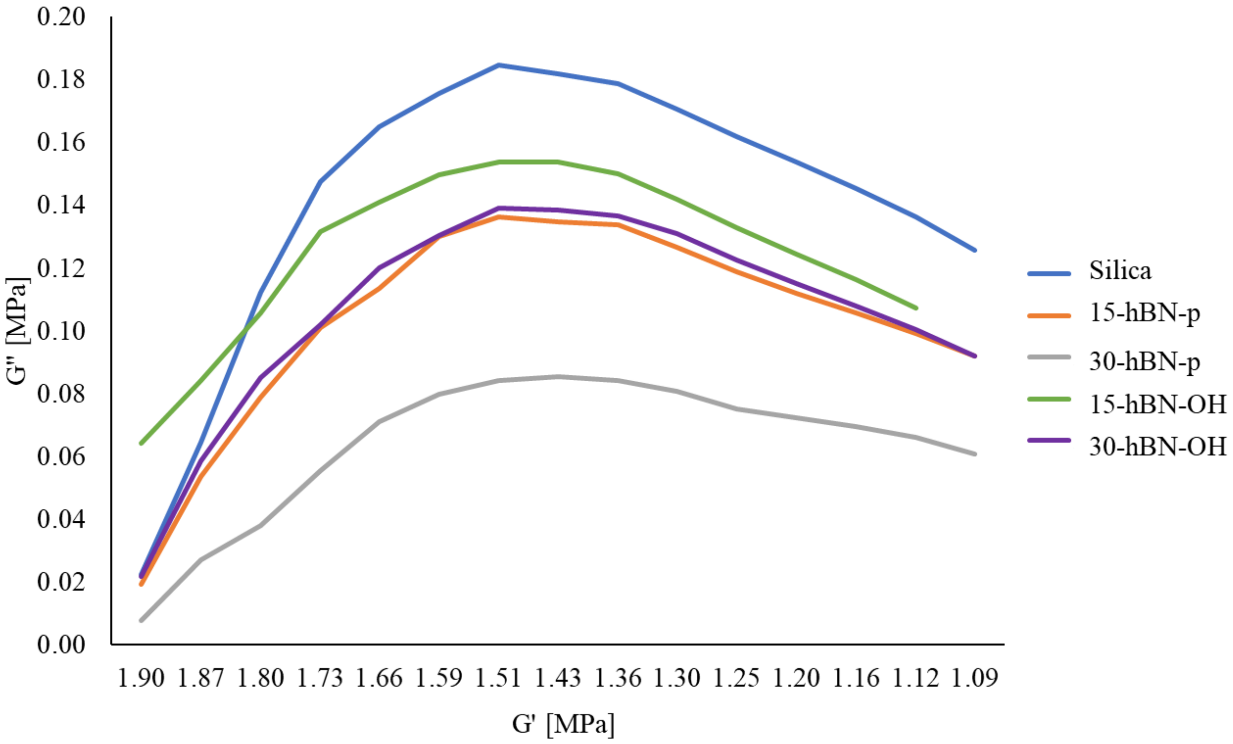

Figure S5. G plot for for composites of Table 1.

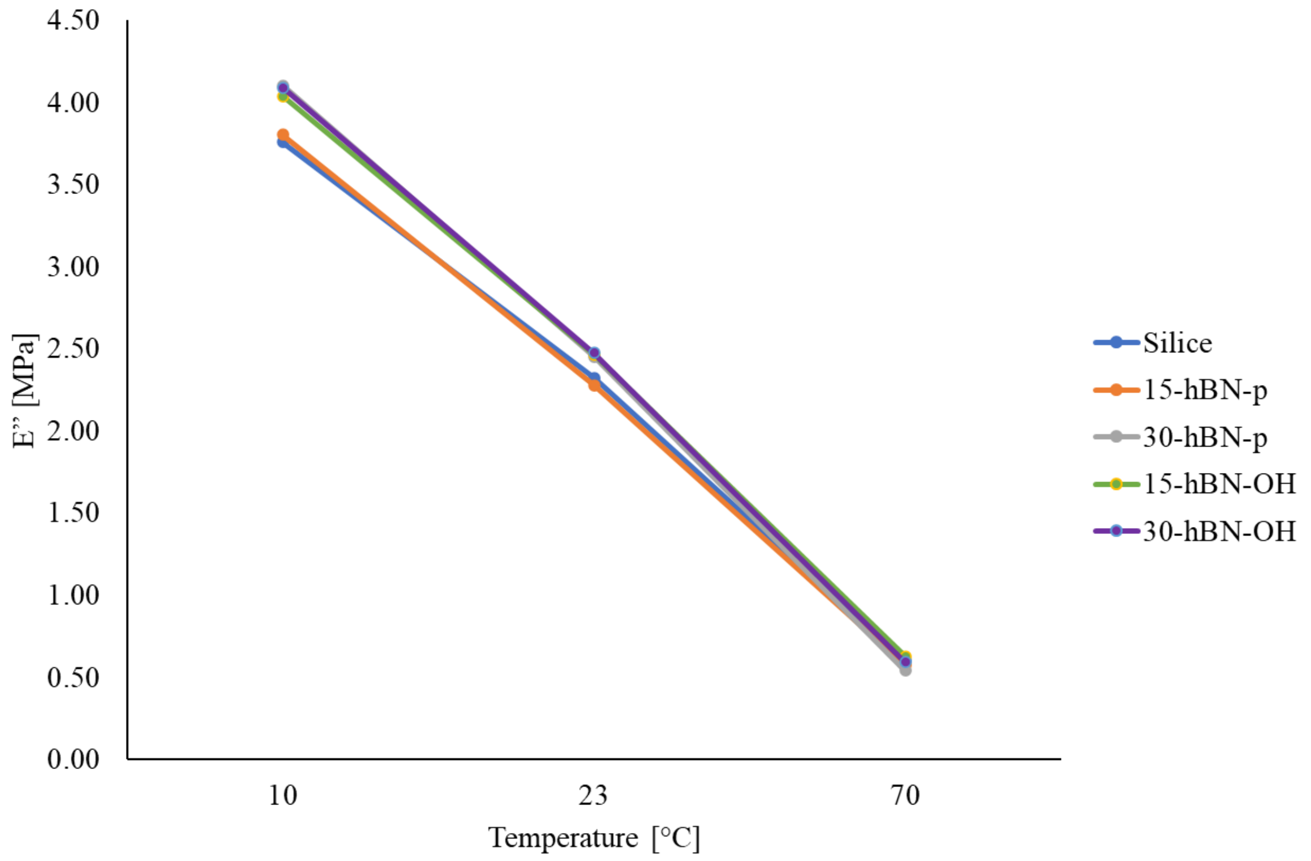

Figure S6. E″ vs Temperature for for composites of Table 1.
